# Influence of rapidly oscillating inspired O_2_ and N_2_ concentrations on pulmonary vascular function and lung fluid balance in healthy adults

**DOI:** 10.3389/fphys.2022.1018057

**Published:** 2022-12-07

**Authors:** Eli F. Kelley, Alex R. Carlson, Robert J. Wentz, Briana L. Ziegler, Bruce D. Johnson

**Affiliations:** ^1^ AFRL, 711HPW, WPAFB, Dayton, OH, United States; ^2^ Department of Cardiovascular Diseases, Mayo Clinic, Rochester, MN, United States

**Keywords:** oxygen oscillations, pulmonary vascular, lung fluid balance, pulmonary-capillary blood volume, alveolar capillary barrier

## Abstract

**Introduction:** Aircrew may experience rapidly oscillating inspired O2/N2 ratios owing to fluctuations in the on-board oxygen delivery systems (OBOG). Recent investigations suggest these oscillations may contribute to the constellation of physiologic events in aircrew of high-performance aircraft. Therefore, the purpose of this study was to determine whether these “operationally-relevant” environmental challenges may cause decrements in measures of pulmonary vascular physiology.

**Methods:** Thirty healthy participants (Age: 29 ± 5 years) were recruited and assigned to one of the three exposures. Participants were instrumented for physiologic monitoring and underwent baseline cardiopulmonary physiology testing (ground level) consisting of a rebreathe method for quantifying pulmonary blood flow (Qc), pulmonary capillary blood volume (Vc) and alveolar–capillary conductance (Dm). Ultrasound was used to quantify “comet tails” (measure of lung fluid balance). After baseline testing, the participants had two 45 min exposures to an altitude of 8,000 ft where they breathed from gas mixtures alternating between 80/20 and 30/70 O_2_/N_2_ ratios at the required frequency (30 s, 60 s, or 120 s), separated by repeat baseline measure. Immediately and 45 min after the second exposure, baseline measures were repeated.

**Results:** We observed no changes in Qc, Dm or Vc during the 60 s exposures. In response to the 30 s oscillation exposure, there was a significantly reduced Qc and Vc at the post-testing period (*p* = 0.03). Additionally, exposure to the 120 s oscillations resulted in a significant decrease in Vc at the recovery testing period and an increase in the Dm/Vc ratio at both the post and recovery period (*p* < 0.01). Additionally, we observed no changes in the number of comet tails.

**Conclusion:** These data suggest “operationally-relevant” changes in inspired gas concentrations may cause an acute, albeit mild pulmonary vascular derecruitment, reduced distention and/or mild pulmonary-capillary vasoconstriction, without significant changes in lung fluid balance or respiratory gas exchange. The operational relevance remains less clear, particularly in the setting of additional environmental stressors common during flight (e.g., g forces).

## 1 Introduction

There are growing concerns over the unexpected physiological events among pilots of US Air Force and US Navy high-performance aircraft. Recent investigations dealing with advanced aircrew flight equipment in high-G capable aircraft have revealed a number of significant challenges that may play a role in the occurrence of unexplained physiological events (PEs) (Committee on Armed Services, 2012). The United States Air Force defines a PE as any injury, illness, or abnormal physiological condition experienced by aircrew or others because of the flight environment which may impact pilot health and/or performance. Significant stressors include high levels of inspired oxygen along with variable and rapid changes in oxygen tension along with dry air.

These stressors may predispose pilots to physiologic decompensations and potentially contribute to the constellation of PEs in aircrew of high-performance aircraft. Effective aircrew operations are dependent upon optimal physiologic performance of operators under adverse environments. Based on preliminary findings from USAFSAM, rapid variability in inspired oxygen concentration over windows of time, in a background of mild-moderate altitude sustained for over 45 min may contribute to the “symptoms” observed in pilots. During flight, there are functionally varying inspired oxygen concentrations delivered to the pilots due to fluctuations in the on-board oxygen delivery systems and environmental pressure. There appears to be a combination of exposure variables, e.g., oscillations in the balance of inspired oxygen/nitrogen ratios, how often the transitions occur and the duration these exposures are experienced before mild changes in physiologic function are noted.

Considering the functional oscillations in inspired oxygen experienced during flight, it stands to reason that aircrew may experience windows during which they are hypoxic or hyperoxic. Additionally, the anti-G straining maneuvers (Whitley, 1997) and other breathing patterns undertaken by these aircrew to maintain consciousness and vigilance may also influence end-tidal and arterial CO_2_ concentrations. It must also be considered that this is taking place in the background of variable barometric pressure (depending on cruising altitude and cabin air pressure). This combination of stressors may pose a threat to normal pulmonary-capillary function.

Given their direct exposure to high inspired O_2_ concentrations, alveolar epithelial and alveolar capillary endothelial cells are prone to hyperoxic induced O_2_ free radical mediated injury, including hyperpermeability of the pulmonary microvasculature leading to impaired gas exchange (Idell, 2003; Romashko et al., 2003; Kannan et al., 2006; Shetty et al., 2008). This vulnerability is especially apparent in the cells and structure of the alveolar-capillary membrane (Nagata et al., 2007). As such, normobaric, hyperoxia has been shown to alter the pulmonary microvasculature in healthy subjects, decreasing capillary perfusion and increasing perfusion heterogeneity (Orbegozo Cortés et al., 2015). Further, it has been well-documented that free radicals play an integral role in the progression of inflammation and cell damage, especially in pulmonary tissue (Winrow et al., 1993; Conner and Grisham, 1996; Paola Rosanna and Salvatore, 2012; Mittal et al., 2014). Indeed, lung microvascular endothelium has demonstrated elevated cytokine levels and a shift towards apoptosis following a 72-h exposure to constant, oscillating high inspired O_2_ (Wohlrab et al., 2021). In fact, short-term high inspired O_2_ concentrations may result in reduced lung microvascular density and perfusion, with the response becoming more heterogeneous after 2 h (Donati et al., 2017). This suggests that O_2_ toxicity can evolve from short-term exposure to high inspired O_2_ concentrations. Indeed, reactive oxygen species (ROS) production deriving from the pulmonary-capillary endothelial increased continuously during a 90 min hyperoxic exposure of 70% O_2_ and was shown to increase exponentially with higher O_2_ concentrations (Brueckl et al., 2006). The attendant increase in ROS levels from hyperoxic exposure have been shown to cause hyperpermeability, coagulopathy, and collagen deposition within the alveolar space (Mach et al., 2011). With respect to high-performance aircraft, exposure time, ambient pressure, and inspired O_2_ concentrations determine the cumulative O_2_ dose resulting in toxicity (Mach et al., 2011).

Additionally, hyperoxia has been shown to affect peripheral and pulmonary vasculature. For instance, acute normobaric, hyperoxia has been shown to cause peripheral vasoconstriction, but pulmonary vasodilation (Bärtsch and Gibbs, 2007; Burtscher et al., 2022). Additionally, acute altitude exposure (i.e., ∼8000 ft) has also been shown to upregulate the peripheral chemoreceptors, which may result in increased downstream sympathetic drive (Bärtsch and Gibbs, 2007). An increase in sympathetic activity has been associated with pulmonary artery hypertension, suggesting a functional role of the sympathetic nervous system in the regulation of pulmonary vascular pressures (Vaillancourt et al., 2017). Indeed, recent literature has demonstrated that sympathetic stimulation may increase pulmonary vascular resistance (Kadowitz et al., 1975; Mercurio et al., 2019). Taken together, this suggests that the degree and duration of the hyperoxic assault during altitude exposure effects the pulmonary vascular physiology in a number of ways.

The previous literature regarding the pulmonary vascular effects of high inspired O_2_ concentrations and altitude exposure may induce variable, functional alterations in pulmonary vascular physiology. However, there is little research investigating the effect of hypobaric, hyperoxia on pulmonary vascular function. As such, the purpose of this study was to determine whether “operationally-relevant” environmental challenges may cause acute decrements in pulmonary vascular physiology.

## 2 Methods, assumptions, and procedures

### 2.1 Participants

Thirty healthy participants (Age: 29 ± 5 years) were recruited for this study. Ten participants (*n* = 8 males, *n* = 2 females for 30 s and 120 s oscillations, and *n* = 7 males, *n* = 3 females for 60 s oscillations) were assigned to one of the three exposure limbs in an attempt to achieve a representative sample of the Air Force pilot population. Participants had no known history of cardiac, pulmonary, and/or metabolic disease, and no reported mental or psychological disorders of attention. Each participant completed both exposures except for one participant who did not complete the second exposure (*n* = 30 and *n* = 29, for completion of both exposure and completion of one exposure respectively). The present study conformed to the principles outlined in the Declaration of Helsinki and was approved by the Mayo Clinic Internal Review Board.

### 2.2 Experimental design

To determine the impact of variable inspired O_2_/N_2_ ratios at different oscillation rates (i.e., 30 s, 60 s, and 120 s) in the background of mild altitude (i.e., 8,000 ft) participants visited the laboratory for a screening visit and study visit on separate days. The oscillation rates refer to the amount of time in seconds a subject was breathing the specific gas concentration. A description of the methods and procedures is provided below.

### 2.3 Screening visit

During this visit, participants were assessed for height (Ht), weight (Wt), blood pressure (BP), 12 lead ECG, basic pulmonary function testing (PFT), and complete blood count to rule out anemia.

### 2.4 Hypobaric chamber study visit

Participants presented to the Mayo Clinic Hypobaric Chamber for the study visit. Before initiation of the altitude study visit, a hyperbaric RN provided a safety screening to include: 1) intake and documentation of vital signs; 2) safety screening for prohibited materials; and 3) safety screening for exposure to hyperbaric conditions within the last 24 h. A lung ultrasound was taken (Philips Lumify Ultrasound, Philips Healthcare Systems) utilizing 28 sonographic windows. The participant was instrumented for monitoring purposes with ECG (GE Analytical Instruments), thoracic impedance (Model 2994 THRIM, UFI), and SpO2 (Instrumentation Laboratory).

Baseline testing was conducted in the multiperson triple-lock hypobaric chamber and consisted of rebreathe for pulmonary blood flow, alveolar—capillary conductance, and lung clearance index (Marquette 110 Medical Gas Analyzer Mass Spectrometer, Perkins Elmer; Sievers 280i Nitric Oxide Analyzer, GE Analytical Instruments).

After baseline testing, the chamber was brought to an altitude of 8,000 ft. (International Standard Atmosphere ∼565 mmHg to account for variations in ambient barometric pressure) at a rate of 2,500 ft/min. Participants were seated in an upright position and placed on a mouthpiece attached to a Hans-Rudolph 4,285 Series Switching Valve (Hans Rudolph) capable of switching between open-circuit breathing bags with the 80/20 and 30/70 O_2_/N_2_ ratios at the required frequency (30 s, 60 s, or 120 s). Peripheral O_2_ saturation, impedance, and ECG were monitored continuously, and every 8 min symptoms were assessed. After 45 min of exposure (henceforth referred to as Arm 1), baseline measures were repeated at ground level (i.e., midpoint). This was followed by another 45-min exposure (henceforth referred to as Arm 2), after which repeat baseline measures were obtained, again at ground level (i.e., post). Approximately 45 min after the end of the second exposure, baseline measures were repeated (i.e., recovery). Participants were instructed to remain on the mouthpiece for the duration of the exposure (Supplementary Figure S1).

### 2.5 Measured and computed variables

#### 2.5.1 Hemodynamics

Cardiac output (Q) was measured during the rebreathe technique (see below) *via* assessment of the disappearance rate of C_2_H_2_. C_2_H_2_ is an inert, soluble gas that enters the blood stream *via* pulmonary diffusion but does not bind to hemoglobin. Therefore, the disappearance rate of C_2_H_2_ is proportional to pulmonary blood flow and in participants without lung disease, the pulmonary blood flow is equal to systemic blood flow (Hardin et al., 2020). Stroke volume (SV) was then determined *via* continuously monitored heart rate (HR).

The partial pressures of O_2_ and CO_2_ (P_ET_O_2_ and P_ET_CO_2_, respectively) were measured *via* a mass spectrometer (Marquette 110 Medical Gas Analyzer Mass Spectrometer, Perkins Elmer; Sievers 280i Nitric Oxide Analyzer, GE Analytical Instruments) from a sample line placed in the expiratory limb of the experimental breathing circuit. Pulse oxygenation was measured *via* the forehead (Radical 7, Massimo, CA, United States). Heart rate and rhythm was recorded using a single-channel bio-amplifier module (FE132, ADInstruments, NSW, AUS). Additionally, lung ultrasound was taken at the pre and post-testing periods (Philips Lumify Ultrasound, Philips Healthcare Systems) utilizing 28 sonographic windows as defined by Summerfield and Johnson (2013). A comet tail was defined as hyperechoic reflections at regions of high acoustic mismatch, signaling increase fluid content. Comet tail clusters were counted as five comet tails for consistent quantification. All ultrasound measurements were done by the same investigator to ensure inter-subject reliability.

Arterial blood draws were taken during resting breathing on the mouthpiece at the pre, mid, post, and recovery time periods to obtain respiratory exchange equivalent and patterns, P_ET_O_2_, and P_ET_CO_2_ for arterial blood gas (ABG) calculations (MedGraphics Cardiorespiratory Diagnostic System, Medical Graphics Corporation). ABGs were obtained from a canula in the radial artery at the wrist of the non-dominant hand, so as not to interfere with the cognitive tests. This canula was inserted by a Respiratory Therapist and maintained by a Registered Hypobaric Nurse. The following measures were calculated: alveolar-arterial oxygen difference (AaDO_2_), alveolar partial pressure of oxygen (PAO2), and arterial partial pressures of oxygen and carbon dioxide (PaO_2_ and PaCO_2_ respectively).

#### 2.5.2 Membrane diffusing capacity and pulmonary-capillary blood volume

Pulmonary blood flow (Vc) and alveolar—capillary conductance (Dm) were calculated from the lung diffusing capacity for carbon monoxide (DLCO) and the lung diffusing capacity for nitric oxide (DLNO). Lung diffusion capacities were assessed using a rebreathe technique by taking advantage of the diffusion‐limited nature of CO and NO gas (Coffman et al., 2017; Coffman et al., 2018). Briefly, DLCO and DLNO were determined *via* the rate of disappearance of CO and NO, respectively. Following a normal expiration, subjects were switched into a rebreathe bag containing the test gas mixture (9% He, 0.3% C18O, 35% O2, and balance N2) and instructed to nearly empty the bag with each breath for 10 consecutive breaths. The ratio of DLNO to DLCO (termed *α* ratio) has previously been determined as 2.26 (Coffman et al., 2017). Additionally, Dm was normalized to Vc to provide a blood volume‐ independent measure of membrane conductance (Agostoni et al., 2006).

Alveolar-capillary conductance (Dm)

$$Dm=\frac{\alpha \;ratio}{DLNO}$$

Pulmonary blood flow (Vc)

$$Vc=\frac{1}{\theta CO∙\left(\left(\frac{1}{DLCO}\right)-\left(\frac{1}{Dm}\right)\right)}$$

#### 2.5.3 Symptoms

A proprietary scale was developed to evaluate subjective rating of respiratory and cognitive symptoms during the exposure (Supplementary Table S1). Participants were asked to rate their symptoms on a scale from 1–5. As the participants were instructed to remain on the mouthpiece for the duration of the exposure, they were asked to provide their rating by holding up the number of fingers corresponding to their symptomology. Following the exposure, participants were given the opportunity to elaborate on their symptoms.

#### 2.5.4 Statistical approach

The measured and computed variables obtained during the baseline, midpoint, post, and recovery time periods were averaged to provide a single value per time period. Repeated measures analyses of variance (ANOVAs) using an *α* level of 0.05 to determine statistical significance were used to determine the effect of exposure to oscillating O_2_/N_2_ concentrations in the background of mild altitude on gas exchange and hemodynamics. Data obtained during the chamber visit (e.g., symptoms ratings) were analyzed using separate repeated measures ANOVAs. A Tukey’s honest significant difference (HSD) post-hoc comparison was used to investigate differences between the specific testing time periods.

## 3 Results

### 3.1 Subject characteristics, symptoms, and comet tails

While there was variability within oscillation rates, there were no significant differences between the three groups based on oscillation rates (e.g., 30 s oscillations vs. 60 s oscillations) for subject age, height, weight, or BMI (Table 1). Furthermore, there were no significant changes in symptoms ratings for either respiratory or cognitive symptoms across the exposure (Table 2). While it may appear there are interesting, inter-oscillation trends evident in the symptoms recorded, it must be noted any variation from a “0” rating (i.e., no symptoms at all) can be attributed primarily to a single participant in the 30 s and 60 s oscillation rates. On the other hand, there were more participants who rated themselves as having symptoms in the 120 s oscillation group compared with the 30 s and 60 s oscillations, albeit still minimal symptoms. As such, the most robust finding in our symptoms data is that while no participants gave a symptom rating above a 3 (i.e., Moderate), there were more participants within the 120 s oscillation group that rated as having any symptoms in response to the exposure. Additionally, we observed no statistical differences in the number of comet tails observed pre and post exposure (5.9 vs. 7.0, 6.4 vs. 7.6, and 7.1 vs. 9.6 for 30 s, 60 s, and 120 s oscillations respectively). However, there does appear to be a trend towards increased comet tails, the magnitude of which is larger for longer oscillation rates.

**TABLE 1:** Subject characteristics.

	N (females)	Mean	SD
All subjects			
Age (yr)	30 (7)	28.93	5.30
Height (cm)	30 (7)	175.54	10.48
Weight (kg)	30 (7)	76.56	11.21
BMI	30 (7)	24.79	2.34
30 s oscillation subjects			
Age	10 (2)	30.00	6.02
Height	10 (2)	172.76	7.20
Weight	10 (2)	75.61	8.38
BMI	10 (2)	25.34	2.43
60 s oscillation subjects			
Age	9 (3)	27.30	5.06
Height	9 (3)	176.50	14.14
Weight	9 (3)	75.83	15.10
BMI	9 (3)	24.17	2.01
120 s oscillation subjects			
Age	10 (2)	29.50	4.90
Height	10 (2)	177.35	9.43
Weight	10 (2)	78.24	10.10
BMI	10 (2)	24.87	2.64

SD, standard deviation.

**TABLE 2:** Symptoms ratings.

	Testing time (min)											
	0	8	16	24	32	40	45	53	61	69	77	85
30 s Oscillations												
Respiratory symptoms												
Chest tightness	0	0	0	0	0	0	0	0	0	0	0	0
Desire to cough	0	0	0	0	0	0	0	0	0	0.1	0	0
Other	0	0	0	0	0	0	0	0	0	0	0	0
Cognitive symptoms												
Lightheadedness	0	0.1	0.1	0.1	0.1	0.1	0	0	0	0.1	0.1	0.1
Confusion	0	0	0	0	0	0	0	0	0	0	0	0
Vision	0	0.1	0	0	0	0	0	0	0	0	0.1	0
Other	0	0	0	0	0	0	0	0	0	0	0	0
60 s Oscillations												
Respiratory symptoms												
Chest tightness	0	0	0	0	0	0	0	0	0	0	0	0
Desire to cough	0	0	0	0	0	0	0	0	0	0	0	0
Other	0	0	0	0	0	0	0	0	0	0	0	0
Cognitive symptoms												
Lightheadedness	0	0.1	0	0.2	0.3	0.2	0	0	0.1	0	0	0
Confusion	0	0.1	0	0	0	0.1	0	0	0	0	0	0
Vision	0	0	0	0	0	0	0	0	0	0	0	0
Other	0	0.1	0.2	0.1	0.1	0.1	0	0	0.1	0.2	0	0
120 s Oscillations												
Respiratory symptoms												
Chest tightness	0	0	0	0	0	0	0	0	0	0	0	0
Desire to cough	0	0	0.2	0.1	0.2	0.2	0	0	0	0.1	0.1	0
Other	0	0	0	0	0	0	0	0	0	0	0	0
Cognitive symptoms												
Lightheadedness	0.1	0.1	0.2	0.1	0.2	0.4	0	0	0	0.1	0.3	0.3
Confusion	0	0.1	0.1	0.2	0.2	0.1	0	0.1	0	0.2	0.2	0.2
Vision	0	0	0	0	0.1	0.1	0	0	0	0.1	0.1	0
Other	0	0	0.2	0.2	0.3	0.5	0	0	0	0.1	0.2	0.4

### 3.2 Hemodynamics

There were no changes in cardiac output (Q) observed in response to the 60 s or 120 s oscillations at any testing period. However, our cohort did demonstrate a significant reduction in Q at the post-testing period when compared with the pretesting period in response to the 30 s oscillations, a reduction that was not maintained at the recovery period (Table 3).

**TABLE 3:** The influence of 30 s, 60 s, and 120 s oscillations of 80/20 and 30/70 O_2_/N_2_ concentrations on cardiac output.

Testing period	Mean	SD	*p-value*
30 s oscillation rate			
Pre	5.48	1.02	
Mid	5.16	0.98	0.34
Post	4.88	0.83	**<0.05**
Recovery	5.01	0.97	0.11
60 s Oscillation Rate			
Pre	5.39	1.08	
Mid	5.09	1.07	0.69
Post	5.45	1.29	0.99
Recovery	5.29	1.32	0.98
120 s Oscillation Rate			
Pre	5.52	1.04	
Mid	5.31	0.77	0.80
Post	5.31	0.78	0.80
Recovery	5.37	0.93	0.91

SD, standard deviation. Bolded p-values denote a significant difference between that testing period and the pre exposure testing period (p < 0.05).

### 3.3 Membrane diffusing capacity and pulmonary-capillary blood volume

**FIGURE 1:**
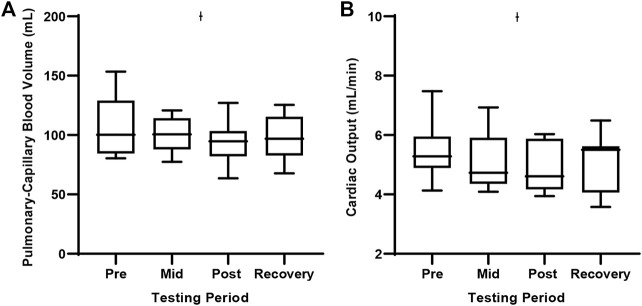
Pulmonary-capillary blood volume and cardiac output during the 30 s oscillation exposure. Values represent means ± SD. **(A)** Depicts pulmonary-capillary blood volume and **(B)** Depicts cardiac output. Ɨ Significant difference from the Pretesting period, *p* < 0.05.

When exposed to the 30 s oscillations, there were no differences in Dm at any testing period in our cohort. Further there was no difference in the Dm to Vc ratio. We observed a significantly reduced Vc at the post-testing period when compared to baseline testing (Table 4; Figure 1). A concomitant reduction in Q at the post-testing period was also observed. Additionally, we observed a systematic decline in Q. It is important to note, we cannot be sure if this reduction in Vc is a result of systemically reduced Q or pulmonary vascular vasoconstriction in response to high inspired O2 concentrations.

**TABLE 4:** The influence of 30 s oscillations of 80/20 and 30/70 O_2_/N_2_ concentrations on lung diffusion measures.

Testing period	Mean	SD	*p-value*
Membrane diffusion capacity (ml/min/mmHg)			
Pre	43.09	8.27	
Mid	42.31	9.13	0.81
Post	43.29	8.19	0.99
Recovery	43.57	9.93	0.99
Pulmonary-capillary blood volume (ml)			
Pre	107.58	25.34	
Mid	100.49	14.98	0.34
Post	94.30	17.86	**0.03**
Recovery	97.47	18.82	0.11
Dm/Vc (1/min/mmHg)			
Pre	0.41	0.08	
Mid	0.45	0.08	0.99
Post	0.49	0.10	0.29
Recovery	0.48	0.12	0.43
Cardiac output (L/min)			
Pre	5.48	1.02	
Mid	5.16	0.98	0.34
Post	4.88	0.83	**0.03**
Recovery	5.01	0.97	0.10

SD, standard deviation. Bolded p-values denote a significant difference between that testing period and the pre exposure testing period (p < 0.05).

**FIGURE 2:**
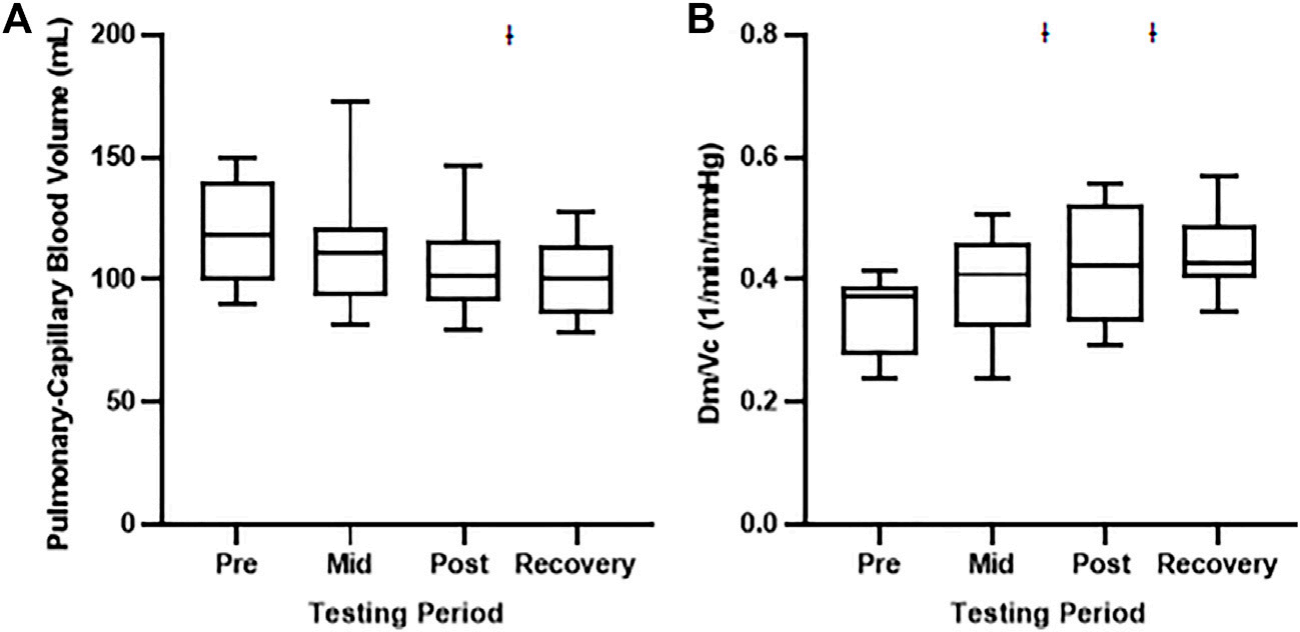
Pulmonary-capillary blood volume and the ratio between membrane diffusion capacity and pulmonary-capillary blood volume during the 120 s oscillation exposure. Values represent means ± SD. **(A)** Depicts pulmonary-capillary blood volume and **(B)** Depicts Dm/Vc ratio. Ɨ Significant difference from the Pretesting period, *p* < 0.01.

During the 60 s oscillations, we observed no changes in any of our gas diffusion or Q parameters (Supplementary Table S2). On the other hand, exposure to the 120 s oscillations resulted in a significant decrease in Vc at the recovery testing period when compared with baseline testing (Table 5; Figure 2). While there was no difference in Dm at any testing period, there was an increase in the ratio of Dm and Vc at both the post and recovery period compared with baseline testing (Table 5; Figure 2).

**TABLE 5:** The influence of 120 s oscillations of 80/20 and 30/70 O2/N2 concentrations on lung diffusion measures.

Testing period	Mean	SD	*p*-value
Membrane diffusion capacity (ml/min/mmHg)			
Pre	38.95	8.09	
Mid	41.43	7.54	0.39
Post	42.88	8.93	0.08
Recovery	42.25	9.88	0.17
Pulmonary-capillary blood volume (ml)			
Pre	119.70	22.18	
Mid	112.16	27.14	0.51
Post	104.97	20.76	0.06
Recovery	100.89	16.00	**<0.01**
Dm/Vc (1/min/mmHg)			
Pre	0.34	0.07	
Mid	0.39	0.09	0.14
Post	0.43	0.09	**<0.01**
Recovery	0.44	0.06	**<0.01**
Cardiac Output (L/min)			
Pre	5.52	1.04	
Mid	5.31	0.77	0.80
Post	5.31	0.78	0.79
Recovery	5.37	0.93	0.90

SD, standard deviation. Bolded p-values denote a significant difference between that testing period and the pre exposure testing period (p < 0.05).

### 3.4 Arterial blood gases

**FIGURE 3:**
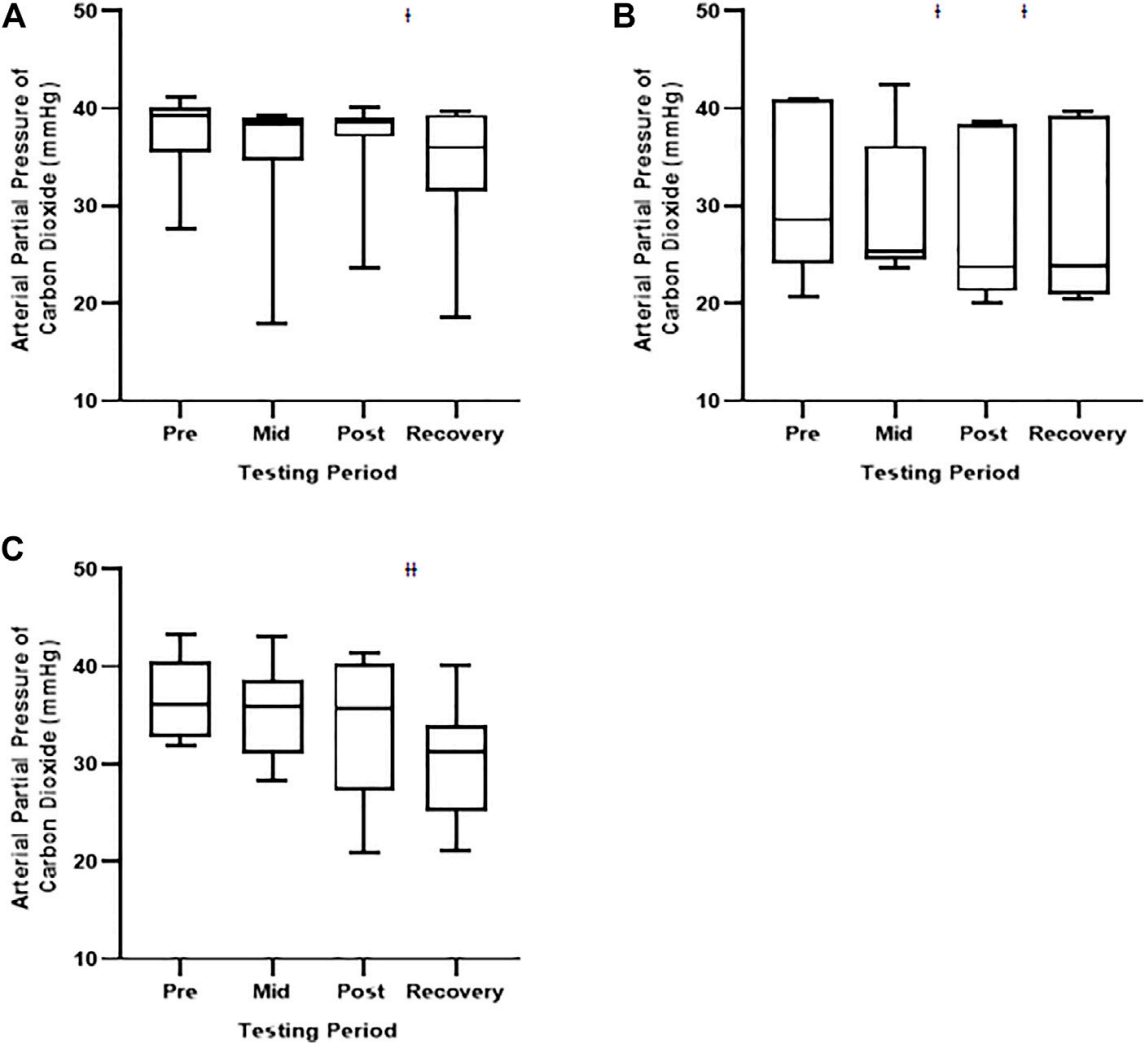
Arterial partial pressure of carbon dioxide during the 30 s, 60 s, and 120 s oscillation exposures. Values represent means ± SD. **(A)** depicts the 30 s oscillations; **(B)** depicts the 60 s oscillations; and **(C)** depicts the 120 s oscillations. Significant difference from the Pretesting period, *p* < 0.05. Ɨ Significant difference from the Pretesting period, *p* < 0.01.

We observed no differences in AaDO_2_ or PaO_2_ between any of the measurement time points during the 30 s, 60 s, nor 120 s oscillation exposures (Tables 6, 7). There were, however, statistically significant reductions in PaCO_2_ for all exposures across time. Specifically, PaCO_2_ was significantly lower at: the recovery testing period in the 30 s exposure cohort; the post and recovery testing period in the 60 s exposure cohort; and the recovery testing period in the 120 s exposure cohort (Table 8; Figure 3). The observed reductions in PaCO_2_ is not surprising given the length of exposure to high inspire O_2_ concentrations.

**TABLE 6:** The influence of 30 s, 60 s, and 120 s oscillations of 80/20 and 30/70 O2/N2 concentrations on alveolar-arterial oxygen difference.

Testing period	Mean	SD	*p*-value
30 s oscillation rate			
Pre	1.04	7.04	
Mid	1.49	9.69	0.99
Post	−2.18	5.09	0.58
Recovery	−2.71	4.99	0.45
60 s Oscillation rate			
Pre	0.16	4.18	
Mid	−2.17	3.29	0.59
Post	−1.52	6.42	0.79
Recovery	−0.03	8.03	0.99
120 s Oscillation Rate			
Pre	2.44	4.84	
Mid	−0.08	3.55	0.69
Post	−1.50	4.06	0.34
Recovery	1.64	6.21	0.98

SD, standard deviation. Bolded p-values denote a significant difference between that testing period and the pre exposure testing period (p < 0.05).

**TABLE 7:** The influence of 30 s, 60 s, and 120 s oscillations of 80/20 and 30/70 O2/N2 concentrations on arterial partial pressure of oxygen.

Testing period	Mean	SD	*p*-value
30 s oscillation rate			
Pre	98.69	15.96	
Mid	104.88	12.67	0.39
Post	102.35	9.88	0.77
Recovery	104.94	17.07	0.38
60 s Oscillation rate			
Pre	113.29	14.78	
Mid	115.93	11.15	0.78
Post	115.71	13.19	0.82
Recovery	114.36	14.72	0.98
120 s Oscillation rate			
Pre	101.13	7.73	
Mid	103.81	9.13	0.94
Post	107.31	13.39	0.57
Recovery	108.19	17.44	0.46

SD, standard deviation. Bolded p-values denote a significant difference between that testing period and the pre exposure testing period (p < 0.05).

**TABLE 8:** The influence of 30 s, 60 s, and 120 s oscillations of 80/20 and 30/70 O2/N2 concentrations on arterial partial pressure of carbon dioxide.

Testing period	Mean	SD	*p*-value
30 s oscillation rate			
Pre	37.51	4.44	
Mid	35.43	7.28	0.31
Post	36.66	5.35	0.89
Recovery	34.08	7.14	**<0.05**
60 s Oscillation Rate			
Pre	31.5	8.51	
Mid	29.97	7.36	0.43
Post	28.34	8.15	**<0.05**
Recovery	28.47	8.39	**<0.05**
120 s Oscillation Rate			
Pre	36.68	4.43	
Mid	35.56	4.84	0.88
Post	33.91	7.37	0.29
Recovery	30.48	5.99	**<0.01**

SD, standard deviation. Bolded *p-values* denote a significant difference between that testing period and the pre exposure testing period (*p* < 0.05).

## 4 Discussion

Taken together, these data suggest “operationally-relevant” environmental challenges may cause acute, albeit mild pulmonary vascular derecruitment, reduced distention and/or mild pulmonary-capillary vasoconstriction, without significant changes in lung fluid balance or respiratory gas exchange. Our data also demonstrated a systemic decline in Q and an associated reduction in Vc. This is not surprising as hyperoxia has been demonstrated to blunt Q (Smit et al., 2018). These data suggest that exposure to shorter O_2_/N_2_ oscillations may have a more pronounced effect on Q compared with longer exposures. The maintenance of Dm and concomitant reduction in Vc resulted in an increased Dm/Vc. This rise in Dm/Vc may indicate shrinkage of the alveolar-capillary membrane, which may be the result of dehydration (Johnson et al., 1960). This may be of particular importance as fighter pilots are especially prone to dehydration, particularly in fifth generation fighter aircraft, which can involve sorties of up to 10 h. These findings suggest exposure to high inspired O_2_ may result in pulmonary-capillary vasoconstriction with little influence on membrane diffusion. However, the observed changes in Vc may also suggest derecruitment due to a small fall in Q or altered vascular tone (reduced NO production or mild hypocapnia).

We propose two mechanisms for these changes in Q or vascular tone. First, we reason these short-term exposures to high inspired O_2_ may present a cumulative effect, especially in the background of mild altitude, may influence inflammatory cell signaling, affecting pulmonary vascular physiology. Indeed, the cells and structure of the alveolar-capillary membrane appear to be especially prone to hyperoxic induced O_2_ free radical mediated injury (Idell, 2003; Romashko et al., 2003; Kannan et al., 2006; Nagata et al., 2007; Shetty et al., 2008). As such, alveolar-capillary units exposed to normobaric, hyperoxia have demonstrated altered pulmonary microvasculature resulting in decreased capillary perfusion and increased perfusion heterogeneity (Orbegozo Cortés et al., 2015). In fact, it has been proposed that short-term exposure to high inspired O_2_ concentrations may engender functional alterations in alveolar-capillary function (Brueckl et al., 2006; Mach et al., 2011).

Secondly, the combination of hyperoxia and altitude exposure may alter the relationship between the peripheral and central chemoreceptors and downstream sympathetic drive. Interestingly, while acute normobaric, hyperoxia exposures have been shown to cause peripheral vasoconstriction, it has also been demonstrated to result in pulmonary vasodilation (Bärtsch and Gibbs, 2007; Burtscher et al., 2022). Additionally, acute, mild altitude exposure has also been shown to upregulate the peripheral chemoreceptors, which may result in pulmonary artery hypertension and vascular resistance owing to increased sympathetic activity (Kadowitz et al., 1975; Bärtsch and Gibbs, 2007; Vaillancourt et al., 2017; Mercurio et al., 2019). As such, previous literature suggests the effects of altitude exposure on pulmonary vascular physiology is largely dependent on the degree and duration of the hyperoxic exposure. In light of these factors, we propose these relatively short oscillation exposures may further increase sympathetic drive, causing increased pulmonary vascular tone.

Given there was no impact on symptoms ratings nor gas exchange, it appears the imposed oscillations in inspired gas concentrations at mild altitude does not constitute a detrimental stressor to pilot pulmonary-capillary function *per se*. However, pilots are exposed to myriad other stressors common during flight, including but not limited to changes in cockpit pressures, G-forces, chest wall restriction, increased respiratory loads, etc. As such, the operational relevance remains less clear in a more operationally relevant setting with additional environmental stressors common during flight.

## 5 Conclusion

The current data demonstrates oscillating inspired gas concentrations at mild altitude alone has little influence on pulmonary-capillary function. However, as an added stressor to those common during flight, it is conceivable that inspired gas concentration oscillation rates and amplitude may engender maladaptive changes in pulmonary-capillary function. As such, minimizing inspired gas concentration oscillation rates and amplitude during flight may prove useful in reducing physiologic events in high-performance aircraft pilots. Given this, consideration should be given to the implementation of pre- and post-sortie testing to evaluate physiologic function and determine if there were any decrements to pulmonary vascular function as a result of said sortie. This may provide more information as to the emergent alterations in pulmonary vascular function engendered by the operational environment and guide protocols for the amount of “down time” prescribed to each pilot to ensure optimal recovery and performance.

## Data Availability

The datasets presented in this article are not readily available because the data used in this manuscript are owned the DoD. Data may be available by contacting the corresponding author and pending USAF STINFO approval. Requests to access the datasets should be directed to EK, eli.kelley@us.af.mil.

## References

[B1] Agostoni P. Bussotti M. Cattadori G. Margutti E. Contini M. Muratori M. (2006). Gas diffusion and alveolar–capillary unit in chronic heart failure. Eur. Heart J. 27 (21), 2538–2543. 10.1093/eurheartj/ehl302 17028107

[B2] Bärtsch P. Gibbs J. S. R. (2007). Effect of altitude on the heart and the lungs. Circulation 116 (19), 2191–2202. 10.1161/CIRCULATIONAHA.106.650796 17984389

[B3] Brueckl C. Kaestle S. Kerem A. Habazettl H. Krombach F. Kuppe H. (2006). Hyperoxia-induced reactive oxygen species formation in pulmonary capillary endothelial cells *in situ* . Am. J. Respir. Cell Mol. Biol. 34 (4), 453–463. 10.1165/rcmb.2005-0223OC 16357365

[B4] Burtscher J. Mallet R. T. Pialoux V. Millet G. P. Burtscher M., (2022)., 0. null, 887–912. 10.1089/ars.2021.0280 Adaptive responses to hypoxia and/or hyperoxia in humans Antioxidants Redox Signal. 0 35102747

[B5] Coffman K. E. Boeker M. G. Carlson A. R. Johnson B. D. (2018). Age‐dependent effects of thoracic and capillary blood volume distribution on pulmonary artery pressure and lung diffusing capacity. Physiol. Rep. 6 (17), e13834. 10.14814/phy2.13834 30175463 PMC6119697

[B6] Coffman K. E. Chase S. C. Taylor B. J. Johnson B. D. (2017). The blood transfer conductance for nitric oxide: Infinite vs. finite *θNO* . Respir. Physiol. Neurobiol. 241, 45–52. 10.1016/j.resp.2016.12.007 28013060 PMC5449205

[B7] Conner E. M. Grisham M. B. (1996). Inflammation, free radicals, and antioxidants. Nutrition 12 (4), 274–277. 10.1016/s0899-9007(96)00000-8 8862535

[B8] Donati A. Damiani E. Zuccari S. Domizi R. Scorcella C. Girardis M. (2017). Effects of short-term hyperoxia on erythropoietin levels and microcirculation in critically ill patients: A prospective observational pilot study. BMC Anesthesiol. 17 (1), 49. 10.1186/s12871-017-0342-2 28335733 PMC5364633

[B9] Committee on Armed Services (2012). F-22 Pilot physiological issues: Hearing before the committee on armed services house of representatives, second sess. (September 13, 2012). Washington: United States Government Publishing Office.

[B10] Hardin E. A. Stoller D. Lawley J. Howden E. J. Hieda M. Pawelczyk J. (2020). Noninvasive assessment of cardiac output: Accuracy and precision of the closed‐circuit acetylene rebreathing technique for cardiac output measurement. J. Am. Heart Assoc. 9 (17), e015794. 10.1161/JAHA.120.015794 32851906 PMC7660774

[B11] Idell S. (2003). Coagulation, fibrinolysis, and fibrin deposition in acute lung injury. Crit. Care Med. 31 (4), S213–S220. 10.1097/01.CCM.0000057846.21303.AB 12682443

[B12] Johnson R. L. Jr. Spicer W. S. Bishop J. M. Forster R. E. (1960). Pulmonary capillary blood volume, flow and diffusing capacity during exercise. J. Appl. Physiol. 15, 893–902. 10.1152/jappl.1960.15.5.893 13790336

[B13] Kadowitz P. J. Joiner P. D. Hyman A. L. (1975). Influence of sympathetic stimulation and vasoactive substances on the canine pulmonary veins. J. Clin. Invest. 56 (2), 354–365. 10.1172/JCI108100 1150876 PMC436594

[B14] Kannan S. Pang H. Foster D. C. Rao Z. WuM. (2006). Human 8-oxoguanine DNA glycosylase increases resistance to hyperoxic cytotoxicity in lung epithelial cells and involvement with altered MAPK activity. Cell Death Differ. 13 (2), 311–323. 10.1038/sj.cdd.4401736 16052235 PMC7091608

[B15] Mach W. J. Thimmesch A. R. Pierce J. T. Pierce J. D. (2011). Consequences of hyperoxia and the toxicity of oxygen in the lung. Nurs. Res. Pract. 2011, 260482. 10.1155/2011/260482 21994818 PMC3169834

[B16] Mercurio V. Pellegrino T. Bosso G. Campi G. Parrella P. Piscopo V. (2019). Cardiac sympathetic dysfunction in pulmonary arterial hypertension: Lesson from left‐sided heart failure. Pulm. Circ. 9 (3), 1–10. 10.1177/2045894019868620 PMC6689920 31328636

[B17] Mittal M. Siddiqui M. R. Tran K. Reddy S. P. Malik A. B. (2014). Reactive oxygen species in inflammation and tissue injury. Antioxid. Redox Signal. 20 (7), 1126–1167. 10.1089/ars.2012.5149 23991888 PMC3929010

[B18] Nagata K. Iwasaki Y. Yamada T. Yuba T. Kono K. Hosogi S. (2007). Overexpression of manganese superoxide dismutase by N-acetylcysteine in hyperoxic lung injury. Respir. Med. 101 (4), 800–807. 10.1016/j.rmed.2006.07.017 17010595

[B19] Orbegozo Cortés D. Puflea F. Donadello K. Taccone F. S. Gottin L. Creteur J. (2015). Normobaric hyperoxia alters the microcirculation in healthy volunteers. Microvasc. Res. 98, 23–28. 10.1016/j.mvr.2014.11.006 25433297

[B20] Paola Rosanna D. Salvatore C. (2012). Reactive oxygen species, inflammation, and lung diseases. Curr. Pharm. Des. 18 (26), 3889–3900. 10.2174/138161212802083716 22632750

[B21] Romashko J. Horowitz S. Franek W. R. Palaia T. Miller E. J. Lin A. (2003). MAPK pathways mediate hyperoxia-induced oncotic cell death in lung epithelial cells. Free Radic. Biol. Med. 35 (8), 978–993. 10.1016/s0891-5849(03)00494-5 14556862

[B22] Shetty S. Padijnayayveetil J. Tucker T. Stankowska D. Idell S. (2008). The fibrinolytic system and the regulation of lung epithelial cell proteolysis, signaling, and cellular viability. Am. J. Physiol. Lung Cell. Mol. Physiol. 295 (6), L967–L975. 10.1152/ajplung.90349.2008 18836029

[B23] Smit B. Smulders Y. M. van der Wouden J. C. Oudemans-van Straaten H. M. Spoelstra-de Man A. M. E. (2018). Hemodynamic effects of acute hyperoxia: Systematic review and meta-analysis. Crit. Care 22 (1), 45. 10.1186/s13054-018-1968-2 29477145 PMC6389225

[B24] Summerfield D. T. Johnson B. D. (2013). “Lung ultrasound comet tails-technique and clinical significance,” in Hot topics in echocardiography (London, UK: IntechOpen), 51–64.

[B25] Vaillancourt M. Chia P. Sarji S. Nguyen J. Hoftman N. Ruffenach G. (2017). Autonomic nervous system involvement in pulmonary arterial hypertension. Respir. Res. 18 (1), 201. 10.1186/s12931-017-0679-6 29202826 PMC5715548

[B26] Whitley P. E. (1997). Pilot performance of the anti-G straining maneuver: Respiratory demands and breathing system effects. Aviat. Space Environ. Med. 68 (4), 312–316. 9096827

[B27] Winrow V. R. Winyard P. G. Morris C. J. Blake D. R. (1993). Free radicals in inflammation: Second messengers and mediators of tissue destruction. Br. Med. Bull. 49 (3), 506–522. 10.1093/oxfordjournals.bmb.a072627 8221019

[B28] Wohlrab P. Johann Danhofer M. Schaubmayr W. Tiboldi A. Krenn K. Markstaller K. (2021). Oxygen conditions oscillating between hypoxia and hyperoxia induce different effects in the pulmonary endothelium compared to constant oxygen conditions. Physiol. Rep. 9 (3), e14590. 10.14814/phy2.14590 33565273 PMC7873712

