# Supplementary material for: Influence of rapidly oscillating inspired O2 and N2 concentrations on pulmonary vascular function and lung fluid balance in healthy adults
Source: Front Physiol. 2022 Dec 7;13:1018057. doi: 10.3389/fphys.2022.1018057 (PMC9768664; doi:10.3389/fphys.2022.1018057)
Supplement: Supplementary file 1 [file Table1.pdf]

Supplementary Table 1. Rating scale to assess respiratory and cognitive symptoms

**Respiratory Symptoms**	
Chest Tightness	
Desire to Cough	0 - None
Other	1 - Little
	2 - Little/Moderate
**Cognitive Symptoms**	3 - Moderate
Lightheaded	4 - Moderate/Severe
Confusion	5 - Severe
Vision Changes	
Other	
