# Supplementary material for: Influence of rapidly oscillating inspired O2 and N2 concentrations on pulmonary vascular function and lung fluid balance in healthy adults
Source: Front Physiol. 2022 Dec 7;13:1018057. doi: 10.3389/fphys.2022.1018057 (PMC9768664; doi:10.3389/fphys.2022.1018057)
Supplement: Supplementary file 2 [file Table2.pdf]

Supplementary Table 2. The influence of 60s oscillations of 80/20 and 30/70 O_2/N_2 concentrations on lung diffusion measures. SD: standard deviation. Bolded *p-values* denote a significant difference between that testing period and the pre exposure testing period ($P < 0.05$).

Testing Period	Mean	SD	*p-value*
**Membrane Diffusion Capacity (mL/min/mmHg)**			
Pre	45.12	7.52	
Mid	47.07	8.32	0.65
Post	46.49	10.65	0.84
Recovery	48.09	8.05	0.30
**Pulmonary-Capillary Blood Volume (mL)**			
Pre	110.85	22.23	
Mid	101.30	18.50	0.11
Post	100.20	17.68	0.06
Recovery	104.08	21.17	0.36
**Dm/Vc (1/min/mmHg)**			
Pre	0.43	0.09	
Mid	0.48	0.08	0.28
Post	0.48	0.07	0.45
Recovery	0.48	0.07	0.27
**Cardiac Output (mL/min)**			
Pre	5.39	1.08	
Mid	5.09	1.07	0.69
Post	5.45	1.29	0.99
Recovery	5.29	1.32	0.98
