# Supplementary figures and images for: Influence of rapidly oscillating inspired O2 and N2 concentrations on pulmonary vascular function and lung fluid balance in healthy adults
Source: Front Physiol. 2022 Dec 7;13:1018057. doi: 10.3389/fphys.2022.1018057 (PMC9768664; doi:10.3389/fphys.2022.1018057)

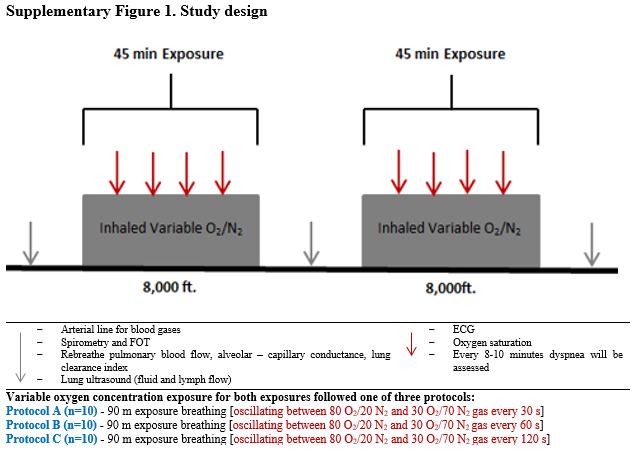

Supplement: Supplementary file 3 [file Image1.TIF]
